# Fracture Severity and Triangular Fibrocartilage Complex Injury in Distal Radius Fractures with or without Osteoporosis

**DOI:** 10.3390/jcm13040992

**Published:** 2024-02-08

**Authors:** Ho-Won Lee, Ki-Tae Kim, Sanghyeon Lee, Joon-Hyeok Yoon, Jung-Youn Kim

**Affiliations:** 1 Department of Orthopedic Surgery, Kangnam Sacred Heart Hospital, Hallym University Medical Center, Seoul 07441, Republic of Korea; lhwghm@gmail.com (H.-W.L.); osgardener@naver.com (S.L.); potter4414@hanmail.net (J.-H.Y.); 2 Department of Orthopedic Surgery, Hallym University Sacred Heart Hospital, College of Medicine, Hallym University, Anyang 14068, Republic of Korea; kimkt8399@naver.com

**Keywords:** distal radius fracture, osteoporosis, displacement of fracture, triangular fibrocartilage complex

## Abstract

The purpose of this study was to investigate the fracture morphology of distal radius fractures (DRFs) with the status of triangular fibrocartilage complex (TFCC) foveal insertion in patients with or without osteoporosis and to identify the relationship between osteoporosis and foveal tear. Seventy-five patients who underwent surgery for DRF from January 2021 to September 2023 were included. All patients were evaluated by standard radiography and dual-energy X-ray absorptiometry and underwent a 3.0 T magnetic-resonance imaging examination of the involved wrist to identify TFCC foveal tear. Patients were allocated into two groups according to the presence of osteoporosis: patients with osteoporosis (group I) and those without osteoporosis (group II). Group I showed a significantly larger displacement of fractures compared to group II (radial inclination; 13.7 ± 5.4 vs. 17.9 ± 4.2; *p* < 0.001, dorsal angulation; 22.2 ± 12.1 vs. 16.5 ± 9.4; *p* = 0.024, ulnar variance; 4.15 ± 2.1 vs. 2.2 ± 1.9; *p* < 0.001). Dorsal angulation and ulnar variance were found to be independent prognostic factors for TFCC foveal tear in logistic regression analysis. Displacement of fractures was related to osteoporosis, and dorsal angulation and ulnar variance were independent prognostic factors for TFCC foveal tear. However, osteoporosis was not identified as a factor associated with TFCC foveal tears.

## 1. Introduction

Distal radius fracture (DRF) is one of the most prevalent fractures, comprising approximately 18% of all fractures, second only to hip fractures in aged people [1]. Concomitant triangular fibrocartilage complex (TFCC) injury is one of the causes of poor clinical outcomes of DRF [2]. It is essential to determine the appropriate treatment approach based on the location and severity of the tear, as traumatic TFCC lesions can lead to distal radioulnar joint (DRUJ) instability, resulting in residual ulnar side pain, decreased grip strength, and restricted range of motion [3]. There is still no clear indication on the management of symptomatic traumatic TFCC injuries; however, they would be managed with immobilization or surgical intervention [3]. Therefore, a preoperative diagnosis of TFCC injuries can assist surgeons in planning an appropriate surgical strategy and rehabilitation protocols.

Anatomical studies have reported that TFCC comprises the distal and proximal components of the radioulnar ligament, originating from the ulnar styloid process and the fovea at the base of the ulnar styloid. Among them, the proximal component consists of a solid ligamentous structure that stabilizes the DRUJ [3]. The insertion site for the proximal component is the fovea ulnaris; therefore, foveal insertion of TFCC plays a vital role in DRUJ stability. The status of the proximal component of TFCC is essential for the additional treatment for a TFCC injury in patients with DRF [4]. Morphological characteristics of DRF are complicated regarding fracture patterns, injury mechanisms, and concomitant ulnar styloid fracture sites, and the fracture severity was related to osteoporosis [5,6]. The morphological characteristics and the fracture severity of distal radius fractures were reported to have a relationship with the TFCC injury; however, limited information is available regarding the relationship between the foveal tear of the TFCC and osteoporosis in patients with DRF.

Diagnosis of the preoperative status of TFCC in DRF patients is challenging [7,8]. The arthroscopic examination is generally regarded as the optimal method to diagnose TFCC tears through direct visualization and probing of the TFCC, with the advantage that treatment can be concomitantly performed using diagnostic arthroscopy [9,10]. However, performing wrist arthroscopy for all DRF patients is difficult due to its invasiveness and potential delay in operation time. Recently, several studies have reported that magnetic resonance imaging (MRI) can be utilized to identify TFCC injuries with excellent intraobserver and interobserver reliability [11,12,13]. The 3.0 T high-resolution MRI makes noninvasive preoperative surgical planning for TFCC lesions possible.

The purpose of the present study was to investigate the fracture morphology such as radial inclination, dorsal angulation, ulnar variance, intra-articular involvement of fracture, and concomitant ulnar styloid base fracture of DRF with the status of TFCC foveal insertion status in patients with or without osteoporosis and to identify the relationship between osteoporosis and foveal tear. We hypothesized that the patients with osteoporosis would exhibit a high prevalence of TFCC foveal tears and severe deformities in DRF, and osteoporosis would be associated with the occurrence of foveal tears in patients with DRF.

## 2. Materials and Methods

### 2.1. Patient Selection

This study retrospectively evaluated patients who underwent surgical treatment for a distal radius fracture at a single institution from January 2021 to September 2023. The inclusion criteria were as follows: patients who underwent open reduction and internal fixation of the distal radius fracture; patients who underwent a preoperative MRI to evaluate TFCC foveal tear. The exclusion criteria were open fractures, skeletal immaturity, loss of preoperative imaging study, arthritis of the wrist or degenerative TFCC tear on the affected wrist, and relevant medical comorbidities affecting the wrist joint. Specific fracture patterns such as partial articular fractures (type B of the AO/OTA classification) [14] and palmarly angulated distal radius fractures were also excluded because of difficulty in evaluating fracture severity. All patients were evaluated by standard radiography and dual-energy X-ray absorptiometry (DEXA) and underwent an MRI examination of the involved wrist using a 3.0 T MRI scanner (Skyra, Siemens, Erlangen, Germany). Osteoporosis was defined as a T-score of −2.5 or less at the lumbar spine, total hip, or femoral neck [15]. Patients were allocated into two groups according to the presence of osteoporosis: patients with osteoporosis (T-score of −2.5 or less) for group I and those without osteoporosis (T-score above −2.5) for group II. All patients completed questionnaires about arm dominance, injury mechanism, and demographics before the surgery, such as age, body mass index (BMI), and sex. The protocol and all procedures of the study were reviewed and approved by the Institutional Review Board of Hallym University Kangnam Sacred Heart Hospital (IRB number: 2023-12-026), and informed consent was obtained from all participants.

### 2.2. Radiologic Evaluations

**Figure 1:**
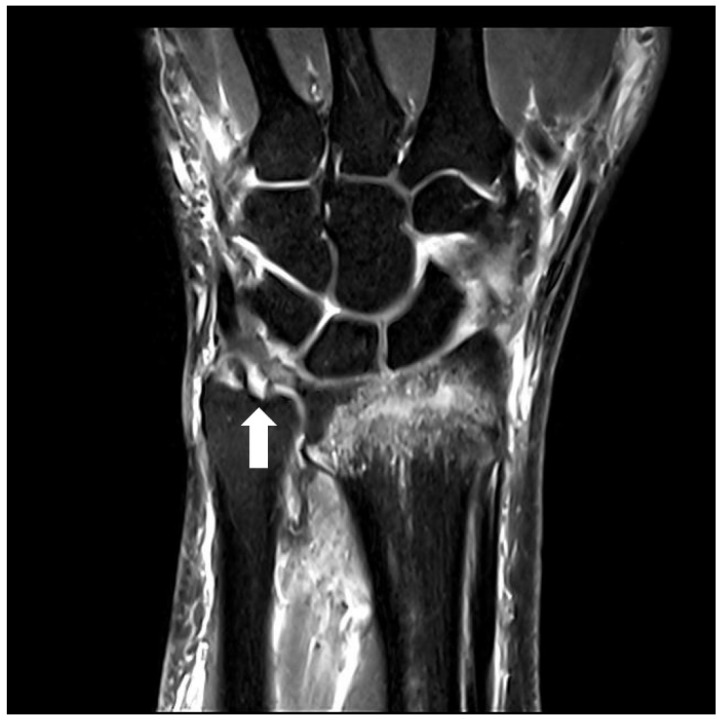
Preoperative magnetic resonance images of the left wrist. Proton density-weighted turbo spin-echo imaging in the coronal plane. The proximal component of the triangular fibrocartilage complex (TFCC) is torn at the foveal attachment (white arrow), compatible with a TFCC foveal tear.

The severity of deformity in distal radius fracture was assessed by measuring the length of ulnar variance, angle of radial inclination, and angle of dorsal angulation at the initial visit and before manual reduction, based on wrist anteroposterior and lateral view of plain radiographs as previously described [16]. Ulnar variance was assessed by the distance between perpendicular lines to the radial axis through the dorsal radial cortex at the radioulnar joint and the distal articular surface of the ulna on the anteroposterior radiograph, while radial inclination was determined by the angle between the perpendicular line to the radial axis and the line joining the distal tip of the radial styloid and the central reference point of distal sigmoid notch on the anteroposterior radiograph [17,18]. The angle of dorsal angulation was measured by the angle created between the perpendicular line to the radial axis and the line joining the most distal points of the dorsal and ventral rims of the distal articular surface of the radius on the lateral radiograph. AO/OTA classification was used in defining the classification and severity of a distal radius fracture [19]. Type A was considered an extra-articular fracture, and Type C was considered an intra-articular fracture. Ulnar styloid fractures were classified based on the fracture line locations. The ulnar styloid status was categorized as intact ulnar styloid, ulnar styloid base fractures, or ulnar styloid tip fractures. Parameters on the plain radiographs were measured using a digital caliper in the Picture Archiving and Communication System by two orthopedic surgeons who were blind to this study, and their measurements were averaged. BMD was measured within one month before the operation in all patients at the lumbar spine and hip using a DEXA scanner (Hologic, Bedford, MA, USA). DEXA for the lumbar spine and the proximal femur was performed by using standard techniques according to the manufacturer and the International Society for Clinical Densitometry guidelines [20]. Preoperative wrist MRI was interpreted by a blinded musculoskeletal radiologist who was not involved in this study. A TFCC foveal tear was defined as a loss of continuity of the foveal fiber or complete bony avulsion with the foveal insertion attached to the fracture fragment (Figure 1) [21].

### 2.3. Statistical Analysis

For continuous variables, the mean differences between the two groups were compared using Student’s *t*-test. To assess differences in proportions between the two groups for categorical variables, the chi-square test or Fisher’s exact test was used. In addition, repeated measurements of clinical scores were evaluated using a paired *t*-test. The level of significance was set at *p* < 0.05 for all tests. Multiple logistic regression analysis was performed to identify independent factors associated with TFCC foveal tears in DRF patients. To identify factors related to TFCC foveal tear, we selected candidate factors that had *p* < 0.1 in the univariate analysis. All statistical analyses were conducted using standard statistical software (SPSS version 26.0, IBM Co., Armonk, NY, USA). Summary statistics are reported as the average values along with their standard deviations for continuous variables, while categorical variables are represented by the count of subjects and their respective percentages.

## 3. Results

**Figure 2:**
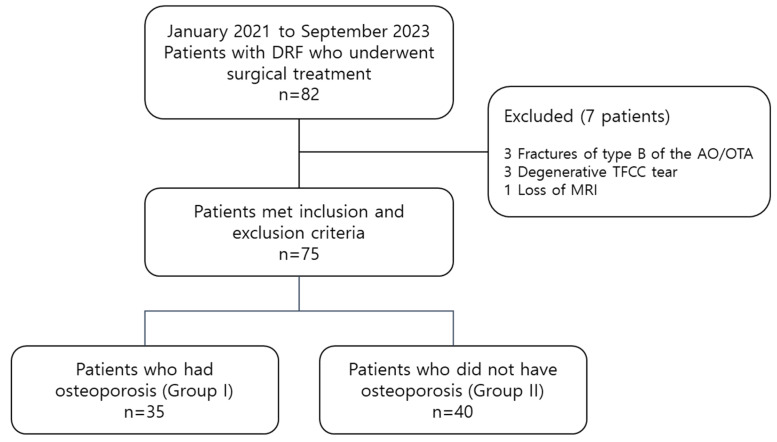
Flow chart describing patient groups and patient selection. DRF, distal radius fracture; TFCC, triangular fibrocartilage complex.

Eighty-two consecutive patients underwent open reduction and internal fixation for a distal radius fracture from January 2021 to September 2023. Of the 82 patients, we excluded 3 patients who had fractures of type B of the AO/OTA classification, 3 with degenerative TFCC tear, and 1 with loss of MRI. Consequently, a total of 75 patients were included in the final analysis (Figure 2).

The mean age of the patients was 63.7 ± 8.8 years; 20 males and 55 females were included in the study. A total of 31 patients (41.3%) injured their wrist on the dominant side, and 49 patients (65.3%) were injured by low-energy trauma such as unintentionally coming to rest on the ground, floor, furniture, or against a wall from a standing height or less. Intra-articular involved fractures were detected in 52 patients (69.3%), and ulnar styloid base fractures were detected in 32 patients (42.7%). The mean radial inclination was 16.6 ± 6.0, the mean angle of dorsal angulation was 19.1 ± 11.1, and the mean ulnar variance was 3.1 ± 2.2, respectively. Associated TFCC foveal tears were detected in 30 patients (40.0%).

Of the 75 patients, 35 patients (46.7%) who had osteoporosis were included in group I, and 40 (53.3%) patients who did not have osteoporosis were included in group II (Table 1). Compared to group II, group I showed a significantly advanced age (*p* < 0.001), a higher proportion of females than males (*p* = 0.005), fractures related to lower energy trauma (*p* = 0.044), radial inclination (*p* < 0.001), angle of dorsal angulation (*p* = 0.024), and ulnar variance (*p* < 0.001). There were no significant differences in the other demographic parameters, including BMI, fracture involvement of the dominant side, and TFCC foveal tear (*p* > 0.05).

Comparisons of fracture patterns between groups are summarized in Table 2. According to the AO/OTA classification for DRF, 2 patients (2.7%) had A2 fractures (extra-articular fracture involving a simple impacted fragment), 21 patients (28.0%) had A3 fractures (extra-articular fracture involving multiple fragments), 4 patients (5.3%) had C1 fractures (simple intra-articular fracture involving a simple fracture configuration in the metaphysis), 38 patients (50.7%) had C2 fractures (simple intra-articular fracture involving multiple fragments in the metaphysis), and 10 patients (13.3%) had C3 fractures (multiple intra-articular fracture lines and fragments in the metaphysis). In total, 29 patients (38.7%) showed no ulnar styloid fracture, and 14 patients (18.7%) showed an ulnar styloid tip fracture. Ulnar styloid base fractures were observed in 32 patients (42.7%). The intra-articular involved fracture was observed in 26 patients (74.3%) in group I and in 26 patients (65.0%) in group II, with no significant difference (*p* = 0.384). The two groups did not show statistically significant differences in the ulnar styloid base fracture (group I; 42.9%, group II; 42.5%, *p* = 0.975)

Dorsal angulation and ulnar variance were associated with TFCC foveal tear in the univariate analysis. Factors with a significance level of *p* < 0.1 in univariate analyses were included in multivariate analysis. The results from the multivariate analysis revealed that dorsal angulation (odds ratio = 1.26, 95% confidence interval = 1.05–1.50, *p* = 0.011) and ulnar variance (odds ratio = 39.91, 95% confidence interval = 3.88–410.60, *p* = 0.002) were found to be independent prognostic factors for TFCC foveal tear (Table 3).

## 4. Discussion

The most important finding in this study was that severe displacement of fractures was accompanied by osteoporosis, and dorsal angulation and ulnar variance were independent prognostic factors for TFCC foveal tear. Contrary to the hypothesis, no significant difference was observed in the frequency of TFCC foveal tears between patients with and without osteoporosis, and osteoporosis was not identified as a factor related to TFCC foveal tears, even though the severity of fracture was different.

Osteoporosis may compromise cortical thickness and reduce cancellous bone density, potentially influencing the degree of deformity in the DRF. Previous studies have reported an association between fracture severity and low bone mineral density (BMD) in patients with DRF [6,22,23]. In addition, Sakai et al. [6] have demonstrated that deformity of fracture, including ulnar variance, radial inclination, and dorsal angulation, was closely associated with bone mineral density of the lumbar spine in patients with DRF. They performed a stepwise regression analysis and found that BMD was significantly related to ulnar variance and that ulnar variance was associated considerably with radial inclination and dorsal angulation. These studies were consistent with our results that osteoporosis diagnosed using the central DEXA was related to the fracture deformity. However, we found no association between osteoporosis and the AO/OTA classification of intra-articular and extra-articular fractures. This finding is in line with previous studies showing no correlation between BMD and the severity of distal radius fractures [24,25,26]. Dhainaut et al. [24] assessed central and hand BMD to investigate the relationship between BMD and AO/OTA classification of DRF. They reported that neither central BMD nor peripheral cortical BMD is a major determinant of the severity of DRF. On the other hand, they did not conduct an analysis of the injury pattern. In our study, there was a higher incidence of fractures resulting from low-energy trauma in osteoporosis patients. Therefore, multiple factors other than osteoporosis, such as localized bone quality, geometry, and injury mechanisms, may be more likely to contribute to the type and severity of fractures.

TFCC maintains the stability of the DRUJ and serves as a shock absorber at the ulnar side of the wrist [3]. Additionally, TFCC provides stability to the DRUJ during the rotational movements of the ulna and allows the carpal bones to move harmoniously with the ulna and DRUJ [7]. The primary mechanism of injury for TFCC and DRUJ is often associated with falling onto an outstretched hand, leading to rotational forces on the forearm, and it may be concurrent with wrist fractures [27]. The results of such type of injury, traumatic injuries to the TFCC, specifically TFCC foveal insertion tear, can induce instability in the DRUJ after injury. Therefore, determining the location and extent of the TFCC tear is crucial for establishing an appropriate treatment approach. [28]

MRI plays a crucial role in the diagnosis of TFCC injuries, as it enables the detection of pathological swelling, intra-articular effusion, and soft tissue damage. These aspects are challenging to discern through other examinations, highlighting the significance of MRI in diagnosing TFCC injuries. MRI is indispensable for the accurate assessment of the complex structures surrounding TFCC. Clinically, when there is suspicion of TFCC injury based on examination findings, conducting MRI examinations is necessary to establish an appropriate treatment plan [29]. Potter et al. [30] reported that MRI examinations exhibit 100% sensitivity, 90% specificity, and 97% accuracy. An accuracy in locating the tear site was reported as high as 92% [31]. Another advantage of MRI is its capability to assess other issues in the wrist simultaneously. It enables the differential diagnosis of various conditions causing ulnar-sided wrist pain, including cartilage degeneration, extensor carpi ulnaris tendinopathy, occult fractures, and ulnar impaction syndrome [32]. In ulnar impaction syndrome, signal changes can be observed in the proximal ulnar aspect of the lunate or ulnar articulating surface, as well as in the proximal ulnar part or the ulnar articulating surface of the triquetrum [33]. Additionally, signal changes may be observed in the ulnar head of the radius from the ulnar impaction syndrome. Therefore, in patients with DRF, preoperative MRI can be utilized to pre-emptively identify these lesions, consider the patient’s medical history, and formulate a treatment plan accordingly. When considering MRI scans, novel imaging techniques such as spectral CT provide an initial overview of pathologies like fractures, enabling an early assessment of the necessity for further MRI scans. Schierenbeck et al. [34] conducted a retrospective imaging study to evaluate the diagnostic performance of a three-material decomposition approach for detecting traumatic bone marrow edema of the extremities on spectral computed tomography. They suggested that the enhanced imaging quality of computed tomography has allowed for the early estimation of the extent of bone marrow changes and other pathologies even before the application of MRI. The 3.0 T MRI examination is valuable for assessing TFCC injuries in patients with distal radius fractures [13,35]. However, caution is warranted with lower-resolution MRIs optimized for knee or spine imaging, as they may risk overlooking crucial information for diagnosing and treating wrist injuries [36].

Tears of the TFCC foveal insertion are frequently accompanied by distal radius fractures and are associated with DRUJ instability [21]. The incidence of TFCC injury in association with distal radius fractures has been documented to vary between 39% and 84% [37]. We identified TFCC foveal tears in 40% of patients with DRF using 3T MRI. A previous study analyzing the relationship between radiologic parameters and TFCC tears found that radial shortening is associated with TFCC injury [35]. Similarly, in the current study, multiple logistic regression analysis revealed that TFCC foveal tear was significantly related to ulnar variance and dorsal angulation. Among these factors, the odds ratio for ulnar variance was notably higher than that for dorsal angulation, indicating a substantial impact on the TFCC foveal tear. The relative shortening and dorsal deviation of the distal radius, leading to increased tension in the peripheral soft tissues on the ulnar side of the wrist, could be highly relevant to the TFCC injury. In addition, previous studies have reported the correlation between TFCC injuries and the extent of dorsal or volar angulation in the fracture [38]. In the cadaveric study, the detached TFCC insertion resulted in increased dorsal angulation [39]. The dorsal angulation of distal radius fractures leads to increased tension in the palmar ligaments that insert into the foveal region, ultimately contributing to palmar injuries within the TFCC complex [40].

Kim et al. [35] have reported that osteoporosis showed significant relevance with the pattern of TFCC injury. Contrary to the previous result, although patients with osteoporosis showed more severe radial deformities and shortening than those without osteoporosis, the frequency of TFCC foveal injuries was higher in the osteoporosis group, but there was no statistically significant difference. The variations in injury mechanisms between the two groups could impact the occurrence of TFCC injuries [28]. Additionally, avulsion fractures in areas like calcaneal tuberosity exhibit peak incidence in elderly patients and are associated with decreased BMD [41,42]. Similarly, in osteoporotic patients, when TFCC injury occurs predominantly on the volar aspect of the forearm during a fall with an outstretched arm, where torsional forces are applied, it is possible that the compromised bone quality may lead to the occurrence of fragile fractures rather than ligament injuries. Further study with a larger sample size and clinical correlation, such as arthroscopic exploration, is needed for the generalizability of these findings.

In summary, while osteoporosis was found to be related to fracture deformity, there was no observed association between osteoporosis and the AO/OTA fracture classifications, suggesting that multiple factors, apart from osteoporosis, may more likely contribute to the type and severity of fractures. In this study, TFCC foveal tear was significantly related to ulnar variance and dorsal angulation, and the odds ratio for ulnar variance was higher than that for dorsal angulation. However, contrary to the hypothesis, no significant difference was found in the frequency of TFCC foveal tears between patients with and without osteoporosis, although the severity of fracture was different. It might be possible that the compromised bone quality may lead to the occurrence of fragile fractures rather than ligament injuries at the time of the injury. A more extensive study with clinical correlation is needed for a broader generalization of these findings. Finally, preoperative MRI can be utilized to preoperatively identify pathologic lesions in complex structures after trauma and formulate a treatment plan accordingly in patients with DRF.

## 5. Limitations

This study has several limitations. First, this study was a retrospective analysis using imaging data and medical records, including a relatively small number of patients. Second, we did not investigate recently identified factors associated with fracture severity, such as glucocorticoid use or vitamin D deficiency [24,43]. Third, we did not establish the overall outcomes after surgery or conduct clinical confirmation of DRUJ stability because this study relied on imaging data analysis using 3T MRI. Fourth, we did not investigate postoperative clinical outcomes. In this study, displacement of fractures was associated with osteoporosis, and dorsal angulation and ulnar variance were independent prognostic factors for TFCC foveal tear. Considering the potential impact of these factors on postoperative clinical outcomes and symptoms, such as ulnar side pain, we plan to conduct further studies to explore their relevance with clinical outcomes. Finally, the utility of MRI for nonspecific wrist pain diminishes as TFCC pathologic findings become more common with advancing age [44]. A correlation exists between TFCC abnormalities and age, regardless of the specific diagnosis. However, we excluded abnormalities related to degeneration by primarily analyzing TFCC foveal tears associated with trauma.

## 6. Conclusions

Displacement of fractures was related to osteoporosis, and dorsal angulation and ulnar variance were independent prognostic factors for TFCC foveal tear. However, no significant difference was observed in the frequency of TFCC foveal tears between patients with and without osteoporosis, and osteoporosis was not identified as a factor related to TFCC foveal tears.

## Figures and Tables

**Table 1:** Comparisons of preoperative demographic factors between patients who had osteoporosis (group I) and those who did not (group II).

	Group I (n = 35)	Group II (n = 40)	*p* Value
Age (years) *	69.1 ± 9.0	59.0 ± 5.3	<0.001
Sex *			
Male (%)	4 (11.4)	16 (40.0)	0.005
Female (%)	31 (88.6)	24 (60.0)	
BMI (kg/m^2^)	24.4 ± 3.6	25.6 ± 4.2	0.185
Involvement of dominant side (%)	13 (37.1)	18 (45.0)	0.423
Low energy trauma (%) *	22 (55.0)	27 (77.1)	0.044
Radial inclination (°) *	13.7 ± 5.4	17.9 ± 4.2	<0.001
Dorsal angulation (°) *	22.2 ± 12.1	16.5 ± 9.4	0.024
Ulnar variance (mm) *	4.15 ± 2.1	2.2 ± 1.9	<0.001
TFCC foveal tear (%) *	17 (48.6)	13 (32.5)	0.156

BMI, body mass index, * statistically significant difference between the two groups.

**Table 2:** Comparisons of fracture patterns according to the AO/OTA classification and ulnar styloid fracture location between patients who had osteoporosis (group I) and those who did not (group II).

	Group I (n = 35)	Group II (n = 40)
AO/OTA classification		
A2	1 (2.9)	1 (2.5)
A3	8 (22.9)	13 (32.5)
C1	2 (5.7)	2 (5.0)
C2	20 (57.1)	18 (45.0)
C3	4 (11.4)	6 (15.0)
Ulnar styloid fracture location		
None	13 (37.1)	16 (40.0)
Tip	7 (20.0)	7 (17.5)
Base	15 (42.9)	17 (42.5)

**Table 3:** Univariate and multiple logistic regression analyses for factors related to triangular fibrocartilage complex foveal tear.

Factors	Univariate Analysis			Multivariate Analysis		
	OR	95% CI	*p* Value	OR	95% CI	*p* Value
Sex	1.34	0.02–7.35	0.887			
Age	1.10	0.82–1.48	0.524			
Involvement of the dominant side	2.89	0.08–10.32	0.175			
BMI	0.55	0.24–1.23	0.145			
Osteoporosis	0.01	0.00–2.31	0.133			
Low energy trauma	0.02	0.00–6.53	0.182			
Intra-articular involvement of fracture	0.18	0.00–58.52	0.565			
Ulnar styloid base fracture	1.68	0.04–74.31	0.790			
Radial inclination	1.09	0.69–1.73	0.705			
Dorsal angulation	1.63	0.97–2.75	0.067	1.26	1.05–1.50	0.011
Ulnar variance	9.81	1.48–64.92	0.038	39.91	3.88–410.60	0.002

OR, odds ratio; CI, confidence interval.

## Data Availability

Data is unavailable due to ethical restrictions.
